# Reviews

**Published:** 1864-04

**Authors:** 


### REVIEWS.

*Dalton's Treatise on Human Physiology—Third Edition—Revised and Enlarged, with two hundred and seventy-three Illustrations. Philadelphia:* BLANCHARD & LEA, 1864.

This book has been received by the profession with great favor from its very first appearance. It has increased in favor with each edition, and now having reached its third revision it stands preëminently the most recent and complete work upon Human Physiology in the English Language, or indeed in any language.

It is offered to the medical profession as a text-book for students, and also as a means of communicating in a condensed form, such new facts and ideas in physiology as have marked the progress of the science within a recent period. Much of the teaching is of great practical importance to the medical man, influencing, in various ways, his views of pathology and therapeutics. Perhaps to the advance of physiological science more than to any other, are we indebted for what we have gained in the knowledge of the nature of many diseases and their proper treatment; in any case, the recent doctrines of physiology, which are founded upon a careful experimental basis, are of interest for the physician who desires to keep pace with the annual advance of his profession, since they indicate the present position and extent, of one of the most progressive departments of medicine.

The improvements and additions which have been introduced into this last edition, we notice by the preface, consist in the incorporation into the text of certain new facts and discoveries, relating mainly to details, which have made their appearance within the last three years. These are mainly the results of experiments of the author with regard to the secretion and properties of the parotid saliva in the human subject, and the quantitative analysis of this fluid by Mr. Perkins; the observations of Prof. Austin Flint, jr., on Stercorine, Cholesterine, and the effects of permanent biliary fistula, and those of Prof. Jeffries Wyman on hare-lip in the median line, from arrest of development. New illustrations have also been introduced which materially increase the value of the book.

No words of commendation can now add to the reputation of this work; it is everywhere known and appreciated. The second edition had become exhausted, and students were for some months unable to obtain a supply. The mere announcement of its appearance will afford the highest pleasure to every student of physiology, while its perusal will insure an understanding of the very latest and most advanced views of the science.

*Ellis' Medical Formulary:* By Robert P. Thomas, M. D.

This book contains a variety of valuable tables, a tabular view of the doses of the principal articles of the materia medica, and most of the important prescriptions employed in modern practice. These prescriptions are presented in condensed form, and advantageously arranged—collected from the various medical works where they have been recorded.

The work possesses attractions for the young physician, and we fear to say for the book what it really deserves, least some young practitioner may infer that we think him at liberty to copy his prescriptions. The only objection we see in the book is the one so common and universal in all published prescriptions; medicines are too much compounded. Fashion and habit control this matter in a great degree, and all who desire to make their prescriptions in the highest style of the art, will have a correct and reliable standard in Ellis' Medical Formulary, by R. P. Thomas. M. D.

*Eleventh Annual Meeting of the Illinois State Medical Society, held in Jacksonville, May 5th,* 1863.

The principal papers presented, were Report on Typhoid Fever, by H. Noble, M. D., of Heyworth, Ill.; on Diseases of the Eye, by E. L. Holmes, M. D., of Chicago; Minor Mental Maladies, by Andrew McFarland, M. D.; Report of Committee on Surgery, by Prof. O. Andrews, of Chicago;

Delayed Union of Fractures, by David Prince, M. D., of Jacksonville; Report of Committee on Prize Essays. These Reports and papers have been carefully prepared, and many of them possess great merit. The volume of Transactions shows an energy and ability very creditable to the profession; though we see that the meeting was not attended by a great number of the physicians of the State. This is accounted for in part, at least, by the absence of many as surgeons and assistant surgeons, in the army. Illinois, evidently, has an efficient, capable, and hard working State Medical Society, which will do much towards advancing the interests of the profession and medical science.

---

*The Nervous and Vascular Connection between the Mother and Fœtus in Utero, By* JOHN O'REILLY, M. D., F. R. C. S. I.; *New York*, ROBERT CRIAGHEAD, 85 *Centre Street*, 1864.

This work consists of an ingenious argument to show that there is a nervous connection between the Mother and Fœtus in Utero. Facts illustrative and demonstrative are introduced, and the philosophy of mental impressions being conveyed to the fœtus in utero by the mother is presented according to the original and enlightened views of the author. It is impossible to give our readers any very satisfactory *resume* of the arguments presented or of the opinions entertained by Dr. O'Reilly. He has studied this subject with great care, and is explaining facts which have been hitherto stupidly denied, because they could not be satisfactorily explained. Physicians who deny that mental impressions made upon the mother can be conveyed to the fœtus in utero, are especially invited to read this book; they will see that there are some "stranger things than dreampt of, in their philosophy." We have been deeply interested in the perusal of the book, and hough, perhaps, all the positions are not yet fully established, yet the originality, philosophy, and perspicuity of the work commends it to the careful consideration of all.

---

*Glanders; by* ROBERT JENNINGS, *Veterinary Surgeon, Professor of Veterinary Medicine and Surgery in the Veterinary College of Philadelphia, author of the "Horse and his Diseases," "Cattle and thetr Diseases," etc., etc.*

He says: "The sales of condemned Government horses in New Jersey and adjacent States having introduced into our country and immediate vicinity, the terrible scourge known as "Glanders," I conceive it my imperative duty to warn you in time, of the danger which is threatening our community.

If the experience of a Veterinary Surgeon, who has devoted eighteen years of his life to the relief of the noblest animal in the gift of Providence, is not sufficient to excite your sympathy and even your fears, I am convinced that the following reports, based upon evidential facts and undoubted authority, will attain that object.

You will see the proofs that "Glanders" is a disease without remedy, positively incurable, *extremely contagious*, easily *communicated to man*, and that every day human life is sacrificed to incredulity and ignorance!

Through the liberality of gentlemen of this country, who deserve our thanks, I am enabled to offer you this small pamphlet, the fruit of my researches and observations. Circulate it among your friends, and by your personal exertions avert the calamity which is menacing our welfare."

